# Investigation of nano- and microdomains formed by ceramide 1 phosphate in lipid bilayers

**DOI:** 10.1038/s41598-023-45575-5

**Published:** 2023-10-30

**Authors:** Dominik Drabik, Mitja Drab, Samo Penič, Aleš Iglič, Aleksander Czogalla

**Affiliations:** 1 Laboratory of Cytobiochemistry, Faculty of Biotechnology, University of Wroclaw, F. Joliot-Curie 14a, 50-383 Wrocław, Poland; 2 Department of Biomedical Engineering, Faculty of Fundamental Problems of Technology, Pl. Grunwaldzki 13, 50-377 Wrocław, Poland; 3 Laboratory of Physics, Faculty of Electrical Engineering, University of Ljubljana, Tržaška cesta 25, 1000 Ljubljana, Slovenia; 4 Laboratory of Bioelectromagnetics, Faculty of Electrical Engineering, University of Ljubljana, Tržaška cesta 25, 1000 Ljubljana, Slovenia

**Keywords:** Biophysics, Computational biophysics, Membrane biophysics, Membrane structure and assembly, Biochemistry, Lipids, Biological techniques, Biophysical methods, Imaging, Microscopy

## Abstract

Biological membranes are renowned for their intricate complexity, with the formation of membrane domains being pivotal to the successful execution of numerous cellular processes. However, due to their nanoscale characteristics, these domains are often understudied, as the experimental techniques required for quantitative investigation present significant challenges. In this study we employ spot-variation z-scan fluorescence correlation spectroscopy (svzFCS) tailored for artificial lipid vesicles of varying composition and combine this approach with high-resolution imaging. This method has been harnessed to examine the lipid-segregation behavior of distinct types of ceramide-1-phosphate (C1P), a crucial class of signaling molecules, within these membranes. Moreover, we provide a quantitative portrayal of the lipid membranes studied and the domains induced by C1P at both nano and microscales. Given the lack of definitive conclusions from the experimental data obtained, it was supplemented with comprehensive in silico studies—including the analysis of diffusion coefficient via molecular dynamics and domain populations via Monte Carlo simulations. This approach enhanced our insight into the dynamic behavior of these molecules within model lipid membranes, confirming that nano- and microdomains can co-exist in lipid vesicles.

## Introduction

Biological membranes, complex and heterogeneous in nature, are characterized by the presence of lateral domains. Living cell plasma membranes house various dynamic domains, each differing in composition and size, ranging from nanometers to micrometers in diameter. Analogously, coexistence of liquid ordered ($L_o$) and liquid disordered ($L_d$) domains can be reproduced in simplified model membrane systems^1–3^. These domains within living cells are highly dynamic protein-lipid structures usually smaller than 200 nm. Such small domains are usually referred to as nanodomains (nDs), are formed by mutual interactions and can merge into larger raft microdomains (mDs) in response to signalling. Serving as sorting platforms, these raft domains facilitate the selective association of specific molecules, enabling their involvement in numerous cellular processes^4,5^. Artificial lipid membrane systems, such as giant unilamellar vesicles (GUVs), exhibit the formation of similar mDs in a time span of tens of $\upmu$s. However, these systems also contain nDs^6^, defined in this study as any domain smaller than 250 nm along one axis. The presence of such nDs has been confirmed in both giant plasma membrane vesicles^7^ and artificial tertiary GUV systems^8^. This study focuses on membranes containing ceramide-1-phosphate (C1P), a signalling lipid integral to cell proliferation, apoptosis, and inflammatory responses^9,10^. As C1P can induce negative curvature of lipid bilayers and form domains, its phase behaviour is of particular interest. However, existing literature reports on this subject are limited and often contradictory. The segregation of C1P in artificial membranes was either reported unconditionally^11^, it was dependent on ions presence and pH^12,13^ or was shown to not segregate^14,15^. It was also reported that when C1P16:0/d18:1 was fully hydrated it is able to form both mono- and bilayers, despite its anionic charge^13^. This bilayer is of crystalline state in RT, goes into gel phase at 45 $^\circ$C and transitions into fluid phase at 65 $^\circ$C. It is also reported to induce negative membrane curvature generation^13^, similarly as phosphatidic acids. Nevertheless, it is still unclear whether C1P generates segregation due to its structure and interactions with other components of membrane, interaction of ions, or observed segregation is just a results of phase difference. The nature of such domains also remains not sufficiently investigated.

The exploration of nDs dynamics can be done by spot-variation fluorescence correlation spectroscopy (svFCS). It is one of the most promising single molecule approaches to study membrane domain related phenomena, as it was already successfully implemented to in-depth study of the dynamics of cellular plasma membrane organization^16,17^. By plotting diffusion time against the squared waist of the laser of the focal volume, this technique enables the determination of the effective diffusion coefficient ($D_{eff}$) and the intercept of the diffusion time axis ($t_0$)^18,19^. High $t_0$ values correlate with dynamic clustering, impacting free movement^18^. Conversely, negative $t_0$ is associated with meshwork barriers. In this work we introduce a new protocol that combines svFCS with GUVs and high-resolution imaging to study diffusion coefficient determination and nDs presence in freestanding lipid membranes. This combination (svzFCS)^20,21^, offers a calibration-free methodology for studying lipid vesicles. Unlike those protocols our method uses giant unilamellar lipid vesicles (GUVs). This enabled us for the first time to focus on behaviour of lipids in freestanding, virtually planar membranes^22,23^. The obtained results were set together with classical diffusion coefficients calculated from molecular dynamics simulations to provide information about the nature and composition of investigated systems. Additionally, we employed Monte Carlo simulations^24,25^ and compared it with experimental data obtained from microscopy studies to get information about domain dynamics in greater detail.

In this article we combined several unique methods to better understand domain dynamics in lipid membranes and the domain-inducing behaviour of the C1P molecules under study in 1-palmitoyl-2-oleoyl-glycero-3-phosphocholine (POPC) lipid membranes. We investigated the organization and dynamics of ceramide-1-phosphate in POPC lipid membranes by using spot-variation Z-scan FCS to examine the lateral diffusion of various lipid analogs in C1P membranes. We analysed two types of ceramide-1-phosphate: C1P 18:1/d18:1 (C1P18) and C1P 16:0/d18:1 (C1P16). The diffusion coefficients obtained were compared with classical diffusion coefficients from modelled molecular dynamics systems, offering a more detailed interpretation of experimental data. We also used confocal microscopy to visualize and analyse microdomains, correlating these with Monte Carlo simulations of lipid vesicles to gain further insights into domain dynamics. Finally, we employed structured illumination microscopy (Lattice SIM^2^) and stimulated emission depletion (STED) microscopy to delve deeper into domain dynamics. This work employs several unique methods to characterize domain dynamics in lipid membranes hence providing more detailed insight into both dynamics of lipid domains and the domain-inducing behavior of studied C1P molecules.

## Results and discussion

### Diffusion law study with spot-variation FCS

**Figure 1:**
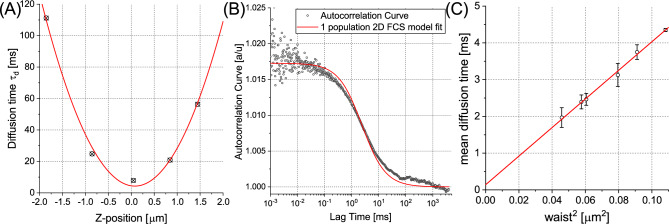
Z-scan approach combined with svFCS. (**A**) Determination of minimal diffusion time $\tau _d$ for a given single POPC vesicle at various z positions at one of waist beam sizes. (**B**) Autocorrelation function used to determine single diffusion time point from previous panel fitted with one population 2D lateral diffusion curve. (**C**) Diffusion law constructed by plotting mean diffusion time for all investigated vesicle at given beam waist size in function of square beam waist size and fitted linearly for POPC GUVs labelled with 1:1000 NBD-Chol.

To delineate the svFCS protocol on GUVs, initial assays were conducted on single-component POPC vesicles labelled with NBD-Chol fluorophore. Significant discrepancies were observed in the diffusion times across a consistent vesicle population, given a constant waist size. This suggested potential complications arising from suboptimal positioning of the point spread function (PSF) relative to the membrane plane, solely considering the maximum fluorescence intensity. To rectify this, we implemented a z-scan strategy wherein the position of peak intensity on the z-axis, as well as two positions above and below it, were identified. The calculated diffusion times were subsequently graphed against the z-position and fitted to a quadratic model (Fig. 1A), thereby procuring the minimum diffusion time, $\tau _d$, for each vesicle. This $\tau _d$ value is deemed the most accurate (Fig. 1B). Following the determination of $\tau _d$ for at least five vesicles, the weighted average and standard deviation were computed, with the weight corresponding to the number of points used in each $\tau _d$ calculation for individual vesicles.

Employing the proposed methodology, we discerned a diffusion law for POPC GUVs as presented in Fig. 1C. Intriguingly, $t_0$ was found to be (0.14 ± 0.12) ms, implying that lateral diffusion in this instance persists unconstrained. The calculated $D_{eff}$ was (6.46 ± 0.11) $\upmu$m$^2$/s, expected to align with *D* as determined in other conventional experiments. Notably, previously documented results suggest slightly higher values of $D=8.87\, \upmu$m$^2$/s at 298 K^26^ (or $D=8.72~\upmu$m$^2/s$ at 293 K calculated with Einstein Relation), a discrepancy potentially attributable to the lower operational temperature of our system. We ruled out the potential influence of agarose on lipid diffusion, in line with previous findings that negate such an effect^27^. Alternatively, it is possible that selected probe contributed to decrease of recorded diffusion coefficient as it was reported that NBD-Chol exhibits local organization even at very low concentration in membranes^28^. Nevertheless, we affirm that the POPC bilayer is homogeneous, and that svFCS serves as an effective tool for probing the presence of domains within lipid membrane systems. Corroborating examples of z-fits and autocorrelation curves for all scrutinized systems are displayed in Figs. S1–S3.

**Figure 2:**
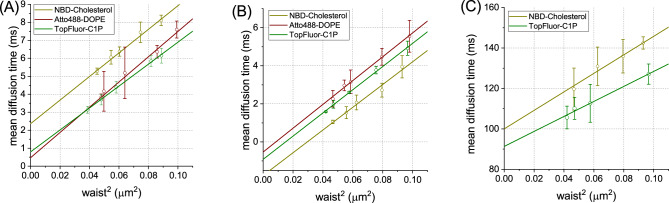
Diffusion laws (mean diffusion time in function of squared beam waist size) obtained for GUVs composed of (**A**) POPC:C1P18 8:2 and (**B**,**C**) POPC:C1P16 8:2. In panel (**B**) diffusion law for faster of the two populations is presented, in panel (**C**) the slower one.

The diffusion behaviour of 8:2 POPC:C1P18 GUVs has been delineated, as illustrated in Fig. 2A. We employed three distinct fluorescent probes in our measurements on C1P-enriched membranes, a methodological choice driven by the known ability of varied fluorescent lipid analogs to differentially modulate membrane dynamics and sorting, or potentially aggregate. By utilizing probes specific to cholesterol, phosphatidylethanolamine, and ceramide 1 phosphate, we mitigated this potential source of bias. The resultant mean effective diffusion coefficient ($D_{eff}$) was determined to be (3.45 ± 0.18) $\upmu$m$^2$/s. Notably, the mean characteristic time ($t_0$) was found to be (1.14 ± 0.42) ms. Detailed results for each individual probe can be found in Supplementary Tables S1 and S2. These findings imply the presence of specific constraints within the POPC:C1P18 lipid bilayer. While phase behaviour of C1P18 remains unexplored in the literature, extrapolations from analogous studies on POPA^29^, suggest a probable $L_o/L_d$ phase coexistence or a similar state in the membrane. Our investigation revealed significant findings for giant unilamellar vesicles (GUVs) incorporating saturated C1P species, notably C1P16, previously documented to induce gel domains^13,30^. The introduction of fluorescently tagged C1P16 has been shown to enhance rigidity and order within the lipid bilayer^31^. Unlike prior observations, autocorrelation curve morphology implies the presence of dual populations, possibly reflecting fluid and gel phases (Fig. S4). This phase-dependent effect on diffusion law aligns with findings from DMPC studies^22^. It is important to note that these two populations were exclusively detected in NBD-Chol and TopFluor-C1P. In contrast, Atto488-DOPE experiments revealed only a single population, indicating divergent phase partitioning among the probes utilized. As such, when feasible, data from the first and second populations were analysed separately. The initial, more rapidly diffusing population yielded a diffusion law depicted in Fig. 2B. The computed mean $D_{eff}$ value was (4.10 ± 0.04) $\upmu$m$^2$/s, analogous to uniform POPC vesicles. Additionally, an intriguing constraint on lipid mobility was noted, yielding an average $t_0$ of (− 0.92 ± 0.23) ms. A negative $t_0$ is indicative of adjacent domains demarcated by barriers, presumably a consequence of a second, slower diffusing population that acts as a barrier and alters the lipid motion in the primary population. Furthermore, the negative $t_0$ could signify the presence of non-spherical barriers, plausible given the morphology of gel domains (e.g., ‘stripes’). Moreover, the induction of gel domains engenders alterations in membrane vertical organization. It has been documented that variations in plasma membrane topography can modulate the observed diffusion time^32^. In our analysis, a secondary, slower-moving population was identified, and a diffusion law was subsequently delineated, as depicted in Fig. 2C. The computed average diffusion coefficient $D_{eff}$ equalled to (0.61 ± 0.05) $\upmu$m$^2$/s. Given that lipid mobility within gel domains is generally much slower^22^, this data suggests that the slower population likely corresponds to the gel phase. The derived $t_0$ value was notably high, reaching (94.4 ± 2.8) ms, implying significant constraints. However, in contrast to prior studies on gel-phase DMPC^22^, a positive $t_0$ value was observed for POPC:C1P16 membranes. This finding suggests that the observed phenomena could not be solely attributed to a phase difference but may also involve a factor of local negative curvature affecting lipid behaviour in membranes. Additionally, it remains plausible that the slower population signifies domain movement rather than lateral lipid diffusion within nDs. To further elucidate the characteristics of the lipid membranes under investigation, molecular dynamics simulations of lipid membrane systems were conducted. These simulations allowed for the comparison of diffusion coefficients in systems with well-defined compositions.

### Molecular dynamics comparison

**Figure 3:**
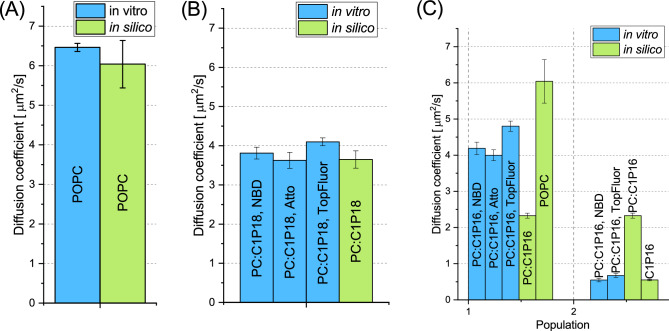
Comparison between $D_{eff}$ obtained from svFCS (blue bars) and respective in silico diffusion coefficient values (green bars) obtained from Molecular Dynamics. (**A**) Diffusion coefficient comparison for POPC membranes. (**B**) Diffusion coefficient comparison for POPC:C1P18 8:2 membranes. (**C**) Diffusion coefficient comparison for POPC:C1P16 8:2 separated depending on the population from svFCS with in silico results from pure POPC, POPC:C1P16 8:2 and pure C1P16 systems.

In our in silico molecular dynamics investigations, we scrutinized the diffusion coefficients derived experimentally, hypothesizing a correlation between lateral diffusion coefficient and membrane composition. The in silico-determined diffusion coefficients serve as membrane composition indicators, aligning with experimental outcomes. Our models included pure C1P16, pure C1P18, POPC:C1P16 8:2, and POPC:C1P18 8:2. The congruity of diffusion coefficients validates similar membrane organization and/or informs about the composition of the examined membrane. As depicted in Fig. 3A, the in silico diffusion coefficient for POPC was reported to equal from (4.8 ± 0.6) $\upmu$m$^2$/s^33^ through 6.04 (after temperature correction)^34^ up to (7.5 ± 0.6) $\upmu$m$^2$/s^35^, to this end we adopted middle ground. Nevertheless, the value of $D_{eff}$ from svzFCS is in agreement with those from in silico reports. It should be noted that described issues with fluorophore are not taken into account when calculating error of $D_{eff}$, so the uncertanity of this parameter might be higher. Similarly, an agreement was found for the POPC:C1P18 mixture with a value of (3.65 ± 0.04) $\upmu$m$^2$/s (Fig. 3B). However, the legitimacy of comparing $D_{eff}$ from svzFCS with in silico simulations requires consideration, particularly when lipid mobility constraints ($t_0 \ne 0$) exist. In a lipid membrane system, such constraints imply the existence of diffusion barriers, likely resulting from lipid segregation. These segregated domains, either nano or macro, are restricted regions that lipids cannot freely enter. The phenomenon of lipid segregation, occurring at a $\upmu$s time regime^36,37^, requires enough lipid molecules for interaction and self-sorting. In contrast, our simulated systems operate within a tens of nanoseconds time regime and contain a significantly lower number of lipid molecules, negating the likelihood of impactful lipid segregation. Therefore, we propose that not only is the comparison of experimental $D_{eff}$ to in silico *D* justified, but it can also be used to infer different diffusion species as observed in FCS. The consistency between these values implies a similarity in composition between in vitro and in silico models. For instance, the diffusion coefficient for the pure C1P18 system was (2.39 ± 0.04) $\upmu$m$^2$/s, indicating a decrease in diffusion coefficient with an increase in C1P18 quantity.

Conversely, the systems with C1P16 exhibit distinct dynamics. The single-component C1P16 membrane simulation yielded D = (0.55 ± 0.02) $\upmu$m$^2$/s (Fig. 3C). In the svzFCS experiment, we observed two diffusion populations, faster and slower. The diffusion rate of the faster population, higher than the in silico POPC:C1P16 8:2 system, was closer to the POPC diffusion coefficient. The in silico value for POPC:C1P16 8:2 was (2.33 ± 0.04) $\upmu$m$^2$/s, while the experimental $D_{eff}$ for the same mixture’s faster population was (4.10 ± 0.04) $\upmu$m$^2$/s. A comparison of the diffusion coefficients for POPC and POPC:C1P16 8:2 indicates that the presence of ceramide reduces the diffusion coefficient, suggesting a predominantly POPC composition with a small fraction of C1P16. As the relationship between diffusion coefficient and local C1P quantity in the membrane remains uncertain, the precise fraction of C1P16 in faster populations cannot be estimated. The slower population’s diffusion aligns with the in silico value for the pure C1P16 system. Interestingly, the second population was undetectable when using Atto488-DOPE as a probe, implying insufficient partitioning to gel nDs although this would require further investigation. In summary, the svzFCS-identified populations closely align with the diffusion coefficients of the single-component C1P16 membrane and the POPC:C1P16 system with a minimal fraction of the second component. This supports the notion that C1P16 forms gel domains coexisting with the fluid phase within the POPC:C1P16 vesicles. Our experimental setup successfully identifies these membrane features. However, the absence of the second population when utilizing Atto488-DOPE as a probe necessitates further exploration. It might suggest that Atto488 DOPE partitioning to gel nDs is not sufficient to be detectable. Yet, this hypothesis requires additional verification through further experimentation, which extends beyond the scope of the present study. Nonetheless, our findings underscore the ability of svzFCS to discern diffusion coefficients of single-component C1P16 membranes and POPC:C1P16 systems with exceptionally low fractions of the secondary component. This further validates the formation of gel domains by C1P16, coexisting with the fluid phase within the POPC:C1P16 vesicles. Consequently, our experimental setup proves effective in identifying these distinct features of the membranes under investigation.

### Visualization and characterization of C1P microdomains

**Figure 4:**
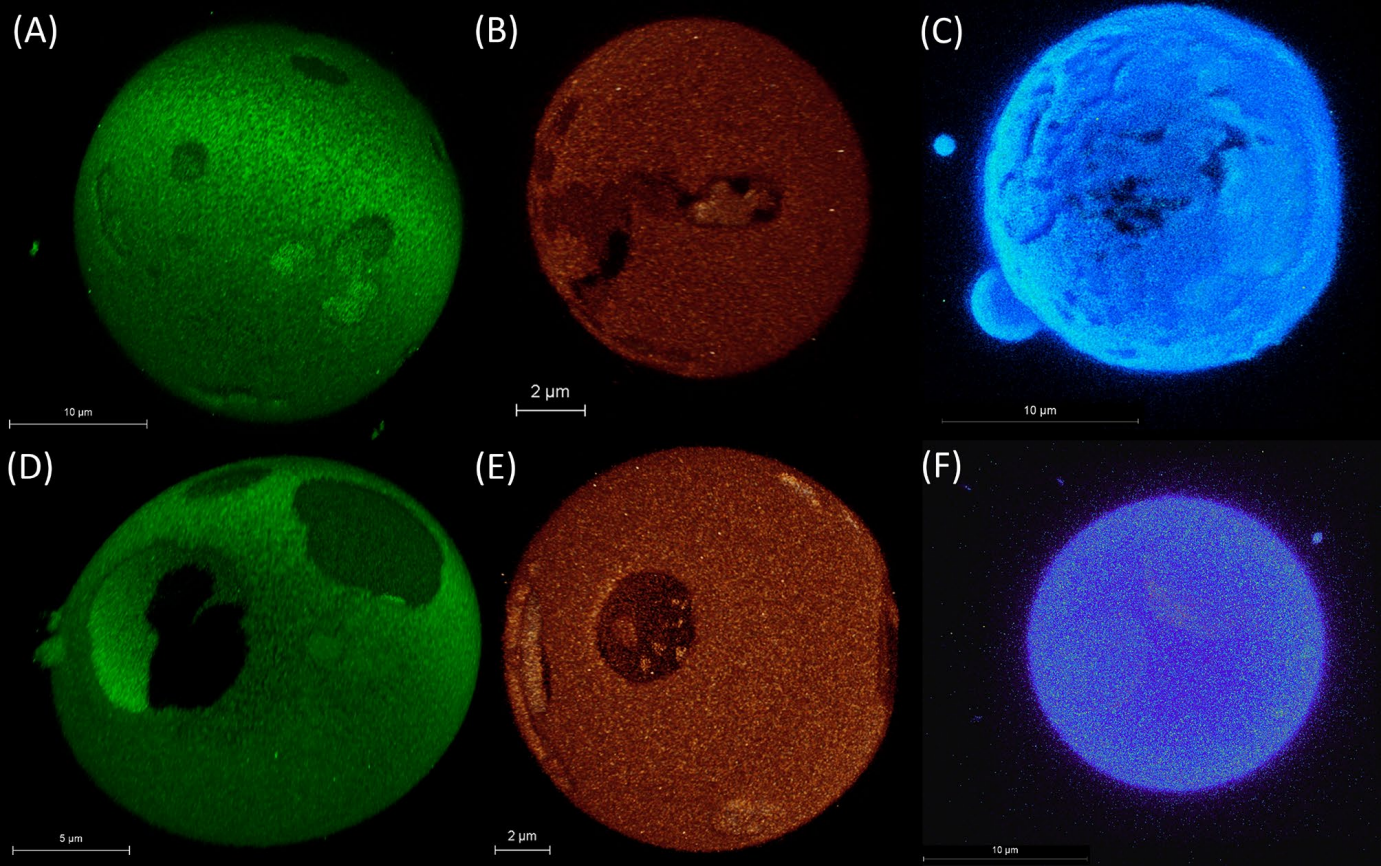
Confocal microscopy Z-stack 3D visualizations of GUVs from POPC:C1P16 8:2 mixture labelled with (**A**) Atto488-DOPE, (**B**) fast DiO, (**C**) TopFluor-C1P and POPC:C1P18 8:2 mixture labelled with (**D**) Atto488-DOPE and (**E**) fast DiO and (**F**) TopFluor-C1P.

Following the identification and approximate delineation of domains within the studied system, we acknowledged the need for further clarification on two issues. Primarily, we had yet to confirm that the observed lipid diffusion constraints were indeed due to the presence of nDs, rather than merely due to mDs serving as physical barriers, which could explain the noted deviations in the $t_0$ values. Moreover, the potential existence of nDs did not inherently validate their stability, raising the possibility of them being meta-stable and coalescing over time into larger microdomains. To address these concerns, our subsequent investigations focused on the visualization and characterization of microdomains, particularly within the POPC:C1P membrane systems known to form larger observable domains within GUVs, as identified by confocal microscopy via areas lacking detectable fluorescent lipid analogs. In order to verify that the observed phase separation was not induced by fluorescent probes, we conducted tests using four probes with varying molecular properties (Atto488-DOPE, Fast DiO, NBD-Chol, and TopFluor-C1P). Phase separation was observed in vesicles containing either C1P16 or C1P18 in all instances, with a single exception. C1P16 vesicles displayed domains with irregular, edged borders and a ‘striped’ topology, indicative of their gel-like nature (Fig. 4A–C). The median domain area across all vesicles was determined to be (0.8 ± 0.6) $\upmu$m$^2$, with a range spanning from 0.05 to 53 $\upmu$m$^2$. It is important to emphasize that the domain area is strongly correlated with the size of the vesicles under investigation. Additional 3D Z-stack visualizations are presented in Fig. S4. Two shape descriptors, roundness and circularity, were utilized in our analysis. In the case of domains observed within the C1P16 system, the mean circularity was 0.298 ± 0.019, and the mean roundness was 0.510 ± 0.083. A comprehensive breakdown of these parameters is provided in Fig. S5.

In the examination of vesicles integrated with C1P18, the domains manifested spherical and circular morphologies, as depicted in Fig. 4D,E. This configuration hints towards more structured yet fluid domains. It should be noted that it is unclear to which extend such membrane is fluid. As there are no sterols in the system, the interplay between molecules can be completely different. Even small change of cholesterol structure lead to significant change in domain properties^38^, so it would be challenging to predict properties of such domains. The median area of these domains was notably larger compared to those observed with C1P16, registering at (9.3 ± 7.9) $\upmu$m$^2$. However, the domains’ areas spanned a wide range, from a minimum of 0.26 $\upmu$m$^2$ to a maximum of 126 $\upmu$m$^2$. A notable outlier was observed in the TopFluor-C1P-labelled vesicles, represented in Fig. 4F. The probe within these vesicles exhibited a uniform distribution within the membranes, which indicates the presence of fluorescently labelled C1P in both domains. More illustrations including 3D Z-stacks and images focusing on NBD-Chol for POPC:C1P16 are available in Fig. S6. The domains observed within the C1P18 system yielded an average circularity of 0.3911 ± 0.034 and an average roundness of 0.634 ± 0.030. As expected, domains associated with C1P16 demonstrated lower roundness. This observation aligns with the theory that the parameter reflecting perimeter length is typically lower for gel domains, given their round and ‘veiny’ morphology. The same pattern holds true for roundness, which is indicative of the sphericity of the examined areas.

Consequently, a significant discrepancy in the shape of the observed domains is apparent between the two systems under investigation. Intriguingly, the presence of microdomains within the POPC:C1P18 system appears to challenge the findings from svzFCS, where no second population was detected in the POPC:C1P18 membrane for the TopFluor-C1P scenario. However, the disparity in diffusion coefficients, derived from in silico simulations for the POPC and POPC:C1P18 systems, is minuscule, around 1 $\upmu$m$^2/s$. It is our contention that this occurrence results from the merging of two similar populations into one on an autocorrelation curve. While it is feasible to accurately ascertain the diffusion coefficient for a single population, it becomes more challenging when two populations with comparable diffusion coefficients coexist. In such instances, the populations become indistinguishable on the autocorrelation curve. A more detailed argument on this matter is provided in Supporting Information Section 3. Nevertheless, the data acquired about domain sizes on vesicles has been employed to validate the accuracy of the Monte Carlo model used for the study of domain dynamics.

### In-silico domain dynamics studied using Monte Carlo model

**Figure 5:**
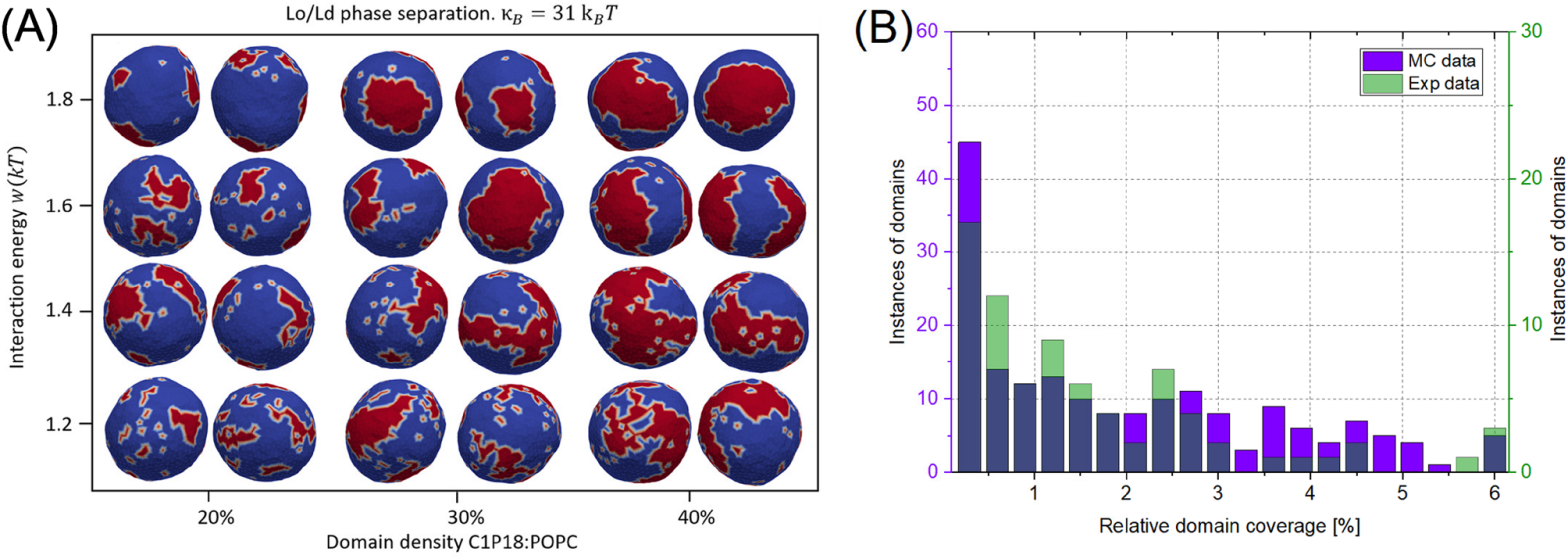
Dynamics of C1P18 domains studied using Monte Carlo model. (**A**) $L_o/L_d$ phase separation snapshots in function of C1P18 concentration (domain density) and interaction energy for POPC:C1P18 model system obtained with Monte Carlo simulations with 1447 vertices. (**B**) Comparison of relative domain coverage from experimental and simulation studies for POPC:C1P18 8:2. Violet and green bars represent excess of either Monte Carlo or experimental data. Dark blue bars represent coverage of both sources (bars).

**Figure 6:**
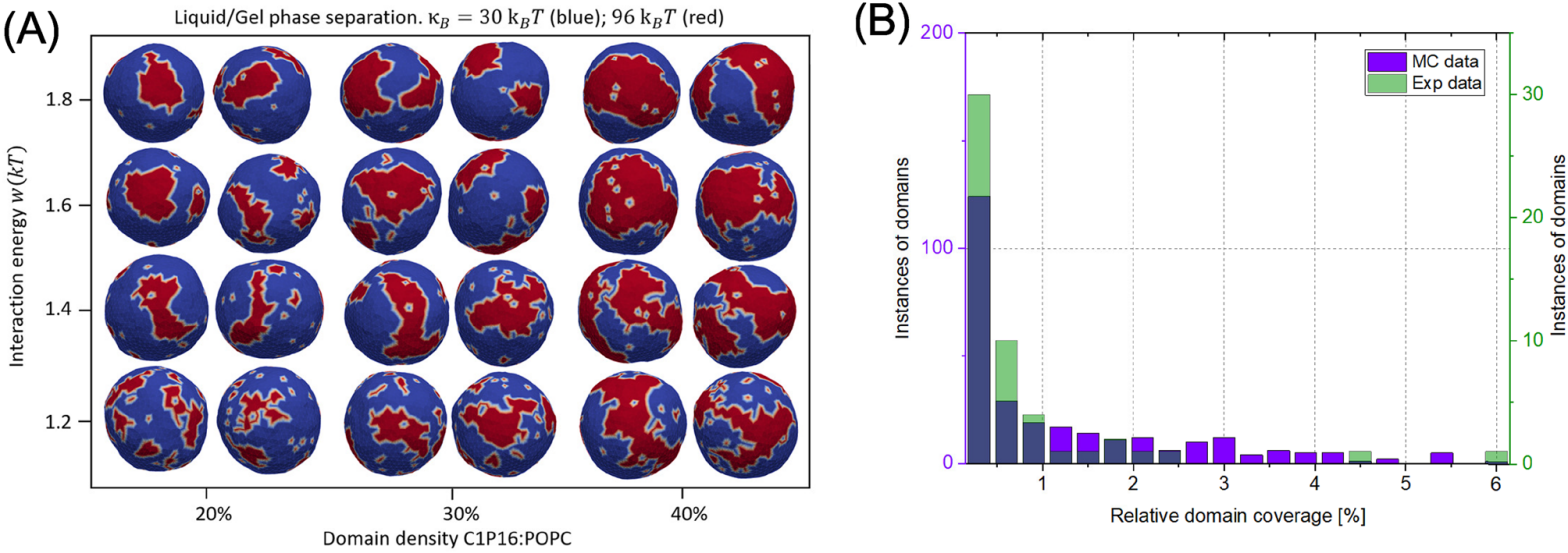
Dynamics of C1P16 domains studied using Monte Carlo model. (**A**) Gel/liquid phase separation snapshots in function of C1P16 concentration for POPC:C1P16 model system obtained with Monte Carlo simulations with 1447 vertices. (**B**) Comparison of relative domain coverage from experimental and simulation studies for POPC:C1P16 8:2. Violet and green bars represent excess of either Monte Carlo or experimental data. Dark blue bars represent coverage of both sources (bars).

While three-dimensional stacks derived from confocal microscopy elucidate the size and characteristics of domains, they do not provide in-depth information regarding domain dynamics or partitioning behavior. Additionally, the investigative scope into the system is circumscribed, primarily due to factors such as light diffraction limitations, which hinder direct observation of nDs. To circumvent these limitations, we utilized a Monte Carlo model of closed vesicles triangulated with a triangular mesh composed of 1447 vertices connected by tethers that form a self-avoiding closed network. The Monte Carlo scheme is based on the minimization of bending energy with a heterogeneous membrane composed of both POPC lipids and C1P domains. The bending rigidity of domains $\kappa$ was obtained from molecular dynamics simulations established with the real-space fluctuation method. Specifically, $\kappa$ was equal to (32.6 ± 0.7) for C1P18 and (96.3 ± 2.2) $k_BT$ for C1P16, respectively. For the POPC membrane, a value of $\kappa$ = 27.3 ± 0.8 $k_BT$ was used, as reported in Ref.^35^. Representative snapshots of Monte Carlo simulations for POPC:C1P18 and POPC:C1P16 models are shown in Figs. 5A and 6A, respectively. In these visualizations, POPC lipids are denoted in blue and C1P domains are depicted in red, while the white color on their boundary is there for better contrast and easier visualization. The area coverage fraction of C1P domains, denoted by $\rho$, increases along the x-axis, while the interaction strength (*w*) between inclusions increases along the y-axis. Our analysis reveals that an increase in w results in the formation of larger C1P domains. The adopted Monte Carlo model leverages a bond flip mechanism to simulate a fluid membrane, with further details provided in supporting material section 5. In instances where domain interaction is absent ($w = 0$), the mixing entropy would yield homogeneously mixed vesicles of blue-red coloration, devoid of apparent clustering. To account for the clustering observed in experimental data, we have incorporated a positive *w* value into our simulations, serving as a variable degree of freedom. We noted that the correspondence with experimental data fluctuated with different *w* values, as shown in Fig. S8.

The dimensionality of identified domains was quantitatively analyzed, and these results were juxtaposed with experimental findings, as illustrated in Fig. 5B. The congruity between in vitro and in silico outcomes was generally commendable, yet disparities were noted in specific domain coverage ranges. In the relative domain coverage interval between 1 and 3, a greater number of instances were discerned in the experimental data. Conversely, the coverage range of 4 up to 4 yielded a larger quantity of instances in the in silico data. These differences may potentially be ascribed to artifacts introduced during the collection of Z-stacks, which could reduce the perceived domain size. The systems under investigation contained a single fluorophore, presenting challenges in accurately delineating domain boundaries. This computational modeling brought two pertinent considerations to the fore. Primarily, the properties of domains ascertained through simulation—namely, their considerable size and circular morphology—provided further corroboration that the lipid segregation observed was reminiscent of *Lo*/*Ld* separation. It merits mention that the Monte Carlo (MC) models contained substantially fewer vertices (N = 1447) compared to experimental data. Consequently, the domain shapes extracted from the MC models may appear more rudimentary in comparison to those obtained through experimental imaging. Yet, the most salient conclusion to be gleaned from these simulations was the concurrent existence of both nDs and mDs. These domains exhibited general temporal stability and did not amalgamate into larger assemblies. In the rare event of such a fusion, a novel ND would segregate from the microdomain elsewhere. Such phenomena was already theoretically described^39^ and was reported to strongly depend on line tension of the system. Interestingly, the approach presented in this work focus on properties of individual domain and probability of separation of nDs from mDs—so local equilibrium based on line tension, while we focused on behavior of whole system and observed that incorporation of nD into mD is followed by separation elsewhere. Nevertheless, it is interesting that two such different approaches resulted in observation of same phenomena.

We conducted analogous simulations for the POPC:C1P16 model, as depicted in Fig. 6A. A distinct characteristic of this system is its gel/liquid state, which influences the morphology of the domains—resulting in sharper edges and less spherical shapes compared to other models. To explore potential variances in vertex interactions within this unique state, we probed the system using three different values of *w*: 1.2, 1.3, and 1.4. The maximum correlation was recorded for $w=1.2$, suggesting comparatively reduced interactions than in the POPC:C1P18 case, where $w=1.4$ was optimal. The histogram depicting the optimal *w* value is shown in Fig. 6B, while the remaining results are presented in Fig. S9. Upon modifying the bending rigidity of the red vertices to mimic that of C1P16, we observed variations in both domain shape and size in our simulations. The domain morphology resembled that of gel domains, characterized by sharp boundaries and numerous stripe-like formations. In addition, we identified the existence of stable nDs within the POPC:C1P16 system, which remained separate over consecutive energy states (MC timesteps) and did not coalesce into larger structures. To investigate whether the mechanical properties of ceramides influence domain shape, we calculated the circularity and roundness of the domains for each system. The corresponding results are illustrated in Fig. S10. Despite exhibiting similar energy and domain density, the ceramides presented different trends. The calculated values for circularity and roundness concurred with experimental results in all instances except for the POPC:C1P16 system. For this system, the experimental data indicated a roundness of 0.51 ± 0.08, whereas the computational data yielded a value of 0.686 ± 0.07. This discrepancy may arise from the more striated shapes of gel domains observed in experimental data compared to those in simulations.

### Nanodomain visualization using SIM^2^ lattice algorithm and $\tau$-STED

**Figure 7:**
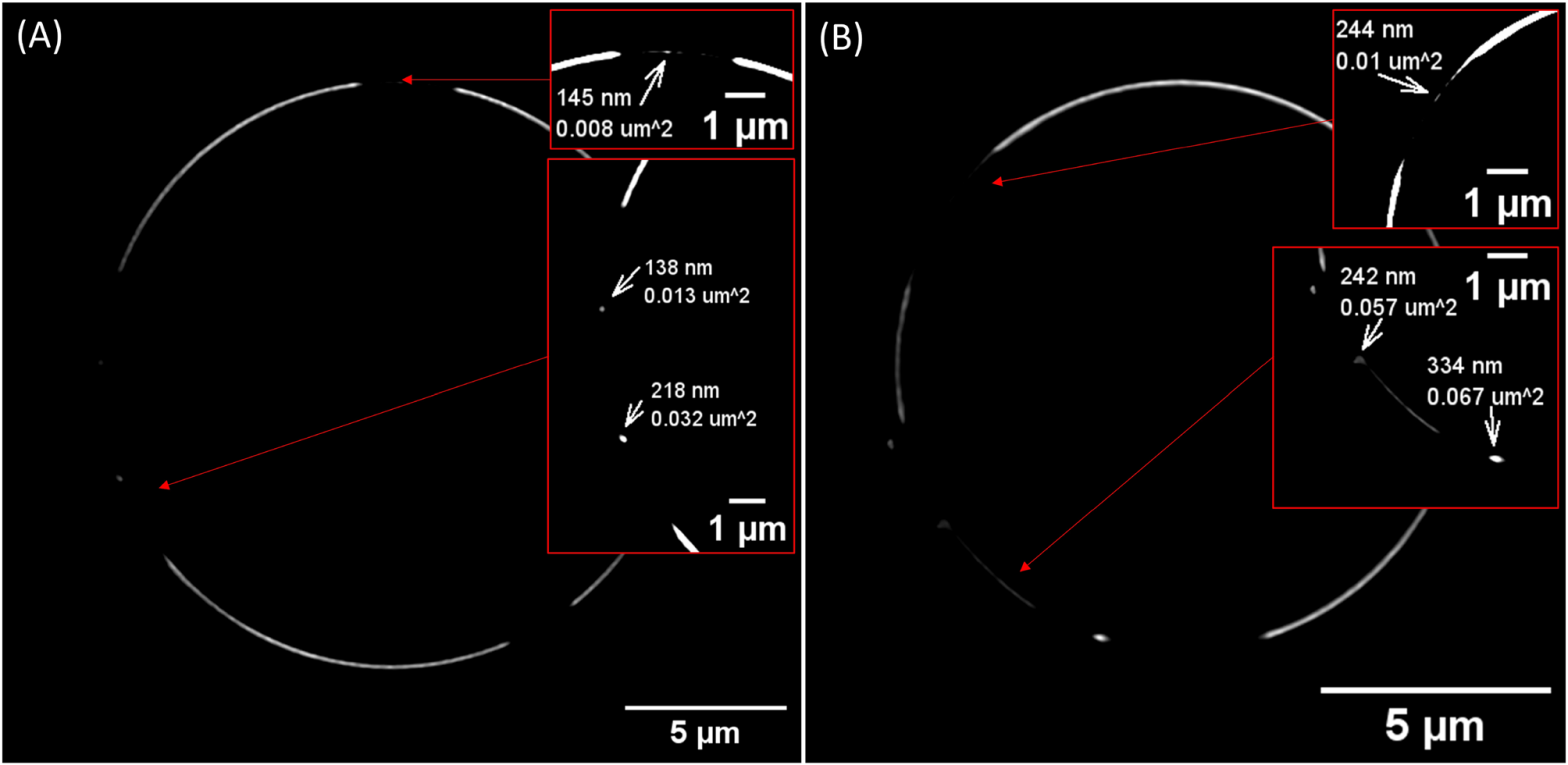
Representative $SIM^2$ images of vesicles of composition. (**A**) POPC:C1P16 8:2 and (**B**) POPC:C1P18 8:2 labelled with Atto488-DOPE with indicated presence of domains along their sizes.

Monte Carlo simulations have established the presence of temporally stable nDs and mDs within the investigated systems. Given the adequate resolution, it is postulated that these nDs can be detected in vitro. For this purpose, we utilized structured illumination microscopy (SIM^2^), capable of discerning structures as small as 60 nm. To minimize the potential for confounding artefacts, our attention was primarily on identifying nDs using fluorescent probes in regions of the vesicle devoid of these probes. Selected micrographs, obtained from both POPC:C1P16 and POPC:C1P18 systems, are depicted in Fig. 7. Although analysing intensity patterns within fluorescent regions of the vesicles could potentially yield further insights about nDs distribution, we opted against this approach due to unequal probe intensity on the control POPC vesicles. As previously outlined in the introduction, we define nDs as any domain smaller than 250 nm in dimension. Therefore, by extension, areas exceeding 300 nm in dimension should be classified as mDs. In adherence to these definitions, we were able to identify points ranging from 160 to 250 nm, suggesting the presence of nDs within this size bracket.

**Figure 8:**
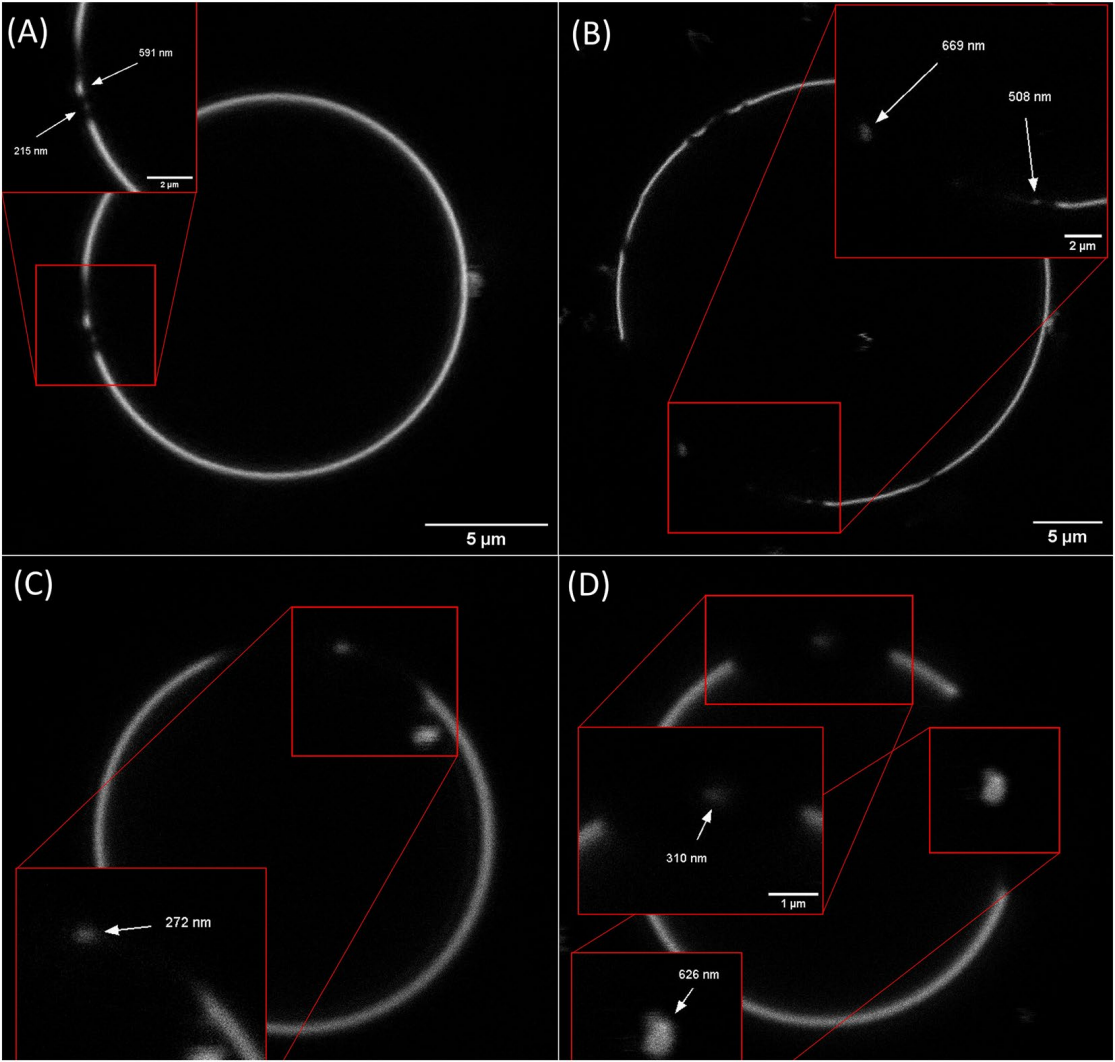
Representative STED images of vesicles. Presence of domains is indicated along their sizes for (**A**,**B**) POPC:C1P16 8:2 and (**C**,**D**) POPC:C1P18 8:2 labelled with Atto550-DMPE.

To further substantiate our observation that the identified objects are not artefacts originating from the SIM^2^ algorithm, we employed stimulated emission depletion (STED) microscopy. In agreement with the SIM^2^ data, STED microscopy also revealed the existence of nDs, as depicted in Fig. 8. We discerned discrete fluorescent regions on the membrane, which varied in their longer diameter, ranging from 215 nm to 670 nm. A subset of these regions fall within the size range characteristic of nDs. It is crucial to note that the acquisition time for STED images exceeds that of SIM^2^, which could potentially lead to the perceptible enlargement of the observed domain sizes. This perceived expansion could be attributed to the dynamic movement of these domains during the extended recording time. The issue of overestimation of domain area in confocal microscopy was highlighted in SLB study^40^. However, in our observations with SIM^2^ this issues should be minimized. It should be noted that an approach to determine sizes of nDs in DOPC/sphingomyelin/cholesterol system was performed^41^. While this report is in agreement with our results regarding dynamics of nDs, they report significantly smaller sizes of nDs (two populations with radii equal to 8 ± 1 and 37 ± 1 nm). Given this report, we cannot exclude that either actual size of nDs formed by C1P is much lower and the area is overestimated due to microscope methodology even with SIM^2^ or C1P domains simply have higher area than the ones made from sphingolipids. Despite that, our findings provide robust and independent confirmation of the presence of NDs.

## Conclusions

In summary, a comprehensive application of both in vitro and in silico techniques was undertaken to scrutinize the lateral organization—encompassing both microdomains (mDs) and nanodomains (nDs)—alongside the dynamics, inclusive of lipid diffusion and temporal evolution, of ceramide-1-phosphate in POPC lipid membranes. This necessitated the development of an innovative methodological framework for the measurement of diffusion law in giant unilamellar vesicles (GUVs) using a spot-variation fluorescence correlation spectroscopy (svFCS) approach enriched with a z-dimension variation. This approach facilitated the extraction of diffusion parameters from vesicles incorporating C1P across three diverse fluorophores. Remarkably, in the control POPC membrane, the measured $t_0$ value approached zero, convincingly indicating an absence of domain constraints or coexistence. In contrast, for both POPC:C1P16 and POPC:C1P18 bilayers, $t_0$ values deviated from zero, thereby suggesting the presence of potential constraints and the probable existence of nDs. Notably, for membranes incorporating C1P16, two distinct populations were discernible in autocorrelation curves. A comparative analysis with in silico derived diffusion coefficients suggested the presence of C1P-dominated nDs and POPC-enriched regions. Conversely, for C1P18, a single population was observed, corroborating the in silico results. To provide a more comprehensive overview, we employed confocal microscopy to elucidate the mDs present in GUVs and investigate their dynamic interactions with nDs. We ensured that the constraints observed in svFCS originated from nDs rather than mDs through a thorough analysis of MD characteristics. Specifically, we utilized confocal microscopy to visualize domain patterns, serving as a reference for parameter optimization in a Monte Carlo simulation model. This model demonstrated that nDs are stable structures. Further, it was evident that the presence of nDs is a consistent feature—as certain nDs amalgamated into larger mDs, while others detached from broader ensembles. Both computational structured illumination microscopy (SIM^2^) and confocal stimulated emission depletion (STED) microscopy revealed the existence of small structures within the investigated GUVs that can be reasonably construed as nDs, thereby affirming the conclusions drawn from the Monte Carlo model. The exploration of the behaviour of various species of C1P, one of pivotal signalling lipid within cells, is crucial to elucidate the plethora of molecular mechanisms modulated by this lipid. Our results indicated the formation of both micro- and nanodomains when C1P was incorporated in the membrane. The heterogeneity of these domains may be instrumental for the optimal recruitment and modulation of proteins involved in cell signalling pathways.

## Methods

### Materials

Lipids POPC (1-palmitoyl-2-oleoyl-glycero-3-phosphocholine), C1P 18:1/d18:1 (N-oleoyl-ceramide-1-phosphate) and C1P 16:0/d18:1 (N-palmitoyl-ceramide-1-phosphate) were purchased from Avanti Polar Lipids (Alabaster, AL, USA). Fluorophores NBD-Cholesterol and TopFluor-C1P were purchased from Avanti Polar Lipids (Alabaster, AL, USA), fast DiO was purchased from ThermoFisher Scientific (Massachusetts, USA) and both Atto550-DMPE and Atto488 DOPE were purchased from Sigma Aldrich (Missouri, USA). Structures of lipids and ceramides are presented in Fig. S11. Doodle of fluorophores showing binding position is presented in Fig. S12. Sucrose and glucose were purchased from Sigma Aldrich (Missouri, USA). Low-melting temperature agarose polymer ($T_m$ 65 $^\circ$C, $T_g$ 25 $^\circ$C) was purchased from Fisher Scientific (Massachusetts, USA). The ultra-pure water used in experiments was obtained from a water purification system (Millipore).

### GUVs electroformation and agarose immobilization

The modified method of model membrane formation for giant unilamellar vesicles (GUVs) was used. Briefly, 10 $\upmu$l of 1 mM POPC:C1P 8:2 mixture and fluorescent probe mixture (0.1mol% for svzFCS, 0.5 mol% for confocal visualization) in chloroform was distributed equally along the platinum electrodes and dried under the vacuum for 1 h. The electrodes were then submerged in aqueous non-conductive sucrose solution (120 mOsm) and a square 10 Hz AC electric field was applied for 10 h with 1 V voltage in a custom PTFE (polytetrafluoroethylene) electro-formation chamber increasing with 1V every hour up to 4V^42^. Agarose immobilization was done according to protocol described by Lira et al.^27^. Briefly, agarose was dissolved in 120 mOsm Glucose solution to obtain either 1 or 2% w/v concentration depending on application. Vesicles and agarose were mixed equally in volume while the polymer was still in the fluid state (around 35–40 $^\circ$C). Final 1% w/v agarose concentration was sufficient for vesicles remaining immobilized for the measurement time in svzFCS, while for visualization 0.5% w/v was applied.

### Spot variation Z-scan fluorescence correlation spectroscopy (svzFCS)

The svzFCS measurements were performed using a custom-made svFCS optical system based on a classical Axiovert 200 M microscope (Carl Zeiss, Oberkochen, Germany) equipped with C-apochromat 40$\times$/1,2 W Corr M27 objective (Zeiss). The protocol was partially based on the one described by Mailfert et al.^16,43^. However, due to changing the investigated sample from cells to GUVs, z-scan approach was implemented to the protocol. The z-scan FCS approach was based on results and conclusions presented by Heinemann et al.^44^ and Benda et al.^45^. The waist size was calibrated with 2 nM Rhodamine 6G solution and 488 nm laser beam illumination at the intensity of 330 $\upmu$W. GUVs analyses were performed at 20 $^\circ$C (293.15 K) while the laser beam was adjusted to 2–4 $\upmu$W. After the vesicle was detected, the point of measurement was set to the centre of the vesicle, which was followed by z-scan to determine the z-position of the upper membrane of the vesicle. There were at least five z-positions for each vesicle (one in the highest intensity z-position, two above and two below). The signal was collected by a series of 6 runs lasting for 10 s each for each z-position of the vesicle. The measurements were carried out on 5 to 8 individual GUVs and the obtained data were analysed by the IGOR Pro software (WaveMetrics). The collected autocorrelation functions were fitted with a 2D lateral diffusion model^20^ (either one or two population depending on the vesicle type) and the mean diffusion time $\tau _d$ was calculated. Five to six waists were analysed in order to construct a single diffusion law. The diffusion law is calculated by the Eq. (1), where $\tau _d$ is the experimentally determined values of diffusion time at different waist $\omega$, and $t_0$ is the extrapolated diffusion time at $\omega ^2=0$. These FCS diffusion laws also allow the determination of an effective diffusion coefficient $D_{eff}$.

1 $$\begin{aligned} \tau _d=\frac{\omega ^2}{4\cdot D_{eff}}+t_0 \end{aligned}$$

### Confocal, SIM^2^ and STED visualization studies

Three-dimensional (3D) z-scans of giant unilamellar vesicles (GUVs) were captured with a Stellaris 8 confocal microscope (Leica, Wetzlar, Germany). This was done using an HC PL APO 86 $\times$/1.20 water immersion objective (Leica). Each image, consisting of 521 $\times$ 521 pixels, was recorded using a hybrid (HyD) detector and a pinhole size of 0.5 Airy units. All fluorophores under investigation were subjected to an incident wavelength of 488 nm. To analyse the domains, we developed a custom script in MATLAB. Initially, we identified the centre position and radius of the vesicle. Then we determined artificial points equally distributed on the sphere of the identified radius to ensure the uniform distribution of points in the pole region. For each point, a rectangular slice was taken from the sphere centre to the point. We then calculated the number of points near the radius and determined the presence of a fluorescence signal based on general quantities accustomed to the given vesicles. Utilizing Fiji shape descriptors, we used the obtained slices to measure the roundness and circularity of the observed domains, as defined by Eqs. (2) and (3), respectively^46^.

2 $$\begin{aligned}{} & {} circularity = (4 \pi \cdot area)/(perimeter^2 ) \end{aligned}$$

3 $$\begin{aligned}{} & {} roundness= (4 \cdot area)/(\pi \cdot (major \_ axis)^2 ) \end{aligned}$$

We employed a Zeiss ELYRA 7 with Lattice SIM^2^ super-resolution microscope (Carl Zeiss, Oberkochen, Germany) to collect SIM^2^ images. This was done using a 63 $\times$, 1.4 NA oil immersion Plan-Apochromat objective lens (Zeiss) at 21 $^\circ$C. We used a 488 nm laser line to illuminate samples and inserted an LBF 405/488/561/642 dichroic mirror into the optical path. Signals were collected by an sCMOS pco-edge 4.2M camera. Raw images (13 phase shifted) were resolved by ZEN black software (Carl Zeiss) with the SIM^2^ Lattice module using Fixed Standard settings. For $\tau$-STED, we used a Leica Stellaris 8 system with an HC PL APO 86 $\times$/1.20 water immersion objective (Leica, Wetzlar, Germany). The selected fluorophore for these measurements was Atto550-DMPE. The white diode emission was set to 554 nm. We recorded images with a hybrid (HyDX) detector and a 0.2 Airy unit pinhole size to enhance contrast and limit the observation to the equatorial plane of vesicles. We applied a 2D-STED donut, which originated from a 660 nm laser. We typically recorded STED images with 20–30 nm pixel size (usually 1024 $\times$ 1024 pixels images) at a speed of 400 Hz and relative intensities of 2% for the white diode and 20% for the STED laser. We analysed the images with a $\tau$-STED approach using the built-in algorithm (with a time-gate from 0 to 1 ns and tau-strength equal to 100).

### Force field modification

C1P molecules did not have pre-existing energy profiles in the Charmm software. To overcome this we adjusted the energy parameters for the lipid head, using the structure of the sphingolipid backbone as a guide. We then built structures for ceramide-1-phosphates with 16 and 18 carbon chains using the CHARMM36 model as a foundation. To represent the fatty acid regions, we used two types of sphingolipids, 18:1/16:0 PSM and 18:1/18:1 OSM. As for the phosphate head region, we based it on the structure of phosphatidic acid (PA) and dioleoylglycerol pyrophosphate (DOPP2), the latter being chosen for its double oxygen bond. We also adjusted the partial charges to align with the Charmm software’s format, as depicted in Fig. S13.

### Molecular dynamics simulations

Moving on to the simulation process, we used software NAMD 2.13 software^47^ to run a detailed simulation of the molecules in motion. The simulation was conducted under fixed conditions of particle number, pressure, and temperature (with CHARMM36 force fields^48^). Lipid membrane systems consisted of 648 lipid molecules (split evenly between two leaflets We added water to the system at a ratio of 75 water molecules per lipid molecule. To balance the charge, we added positive ions to the system. The simulation followed standard procedures for setting up and stabilizing the system. The total time for each simulation was at least 120 ns, with the final 10 ns used for data analysis. The simulations were all conducted at a temperature of 22 $^\circ$C (295.15 K). The snapshots of these systems are shown in Fig. S14. The Diffusion Coefficient Tool plugin was used for lipid molecules lateral (2D) diffusion determination^49^. It was calculated using Einstein’s relation with mean square displacement of phosphorus atoms of the chosen molecular species. Bending rigidity that was used in Monte Carlo was determined using the real-space fluctuation method^50,51^.

### Monte Carlo simulations

The theoretical model for Monte Carlo Simulations is described in details elsewhere^24,25^. Briefly, the membrane is represented by a set of *N* vertices that are linked by tethers of variable length *l* to form a closed, dynamically triangulated, self-avoiding two-dimensional network of approximately 2*N* triangles and with the topology of a sphere^52^. The lengths of the tethers can vary between a minimal and a maximal value, $l_{min}$ and $l_{max}$, respectively. Self-avoidance of the network is ensured by choosing the appropriate values for $l_{max}$ and the maximal displacement of the vertices in a single updating step. The dynamically triangulated network acquires its lateral fluidity from a bond flip mechanism. A single bond flip involves the four vertices of two neighbouring triangles. The tether connecting the two vertices in diagonal direction is cut and re-established between the other two, previously unconnected, vertices. The self-avoidance of the network is implemented by ensuring that no vertex can penetrate through the triangular network and that no bond can cut through another bond.

One Monte Carlo sweep (MCS) consists of individual attempts to displace each of the *N* vertices by a random increment in the sphere with radius *s*, cantered at the vertex, followed by RBN attempts to flip a randomly chosen bond. We denote RB as the bond-flip ratio, which defines how many attempts to flip a bond are made per one attempt to move a vertex in one MCS. Note that the bond-flip ratio is connected to the lateral diffusion coefficient within the membrane, i.e. to the membrane viscosity. In this work we have chosen $R_B = 3$, $s/l_{min} = 0.15$ and $l_{max}/l_{min} = 1.7$. Additionally, an interaction parameter *w* is used in the total triangulated energy expression to reflect the attractive potential between nanodomains:

4 $$\begin{aligned} W_i=-wi\sum _{i<j} H(r_0-r_{ij}) \end{aligned}$$

Here, *H* is the Heaviside step function while $r_0$ is the range of direct interaction and $r_{ij}$ is the in-plane distance between neighboring domains. The dynamically triangulated network acquires its lateral fluidity from the bond-flip mechanism. A single bond-flip involves the four vertices of two neighbouring triangles. The tether connecting the two vertices in diagonal direction is cut and re-established between the other two, previously unconnected, vertices. The self-avoidance of the network is implemented by ensuring that no vertex can penetrate through the triangular network and that no bond can cut through another bond. The microstates of the membrane are sampled according to the Metropolis algorithm. The probability of accepting the change of the microstate due to vertex move or bond-flip is:

5 $$\begin{aligned} min[1,\exp {(-\Delta E/k_BT)}] \end{aligned}$$

where $-\Delta E$ is the energy change, $k_B$ is the Boltzmann constant and T is absolute temperature. The energy for a given microstate is specified by Eq. (1). The bending energy is discretized as described by Gompper and Kroll^52^. For each set of parameters, the system is initially thermalized. Ensemble averaging is done over 200 statistically independent microstates. The values of bending rigidity were taken from MD simulations.

### Statistical analysis

To test the significance of difference between the parameters, unless specified otherwise, the one-way ANOVA test was used with the significance level at 0.05. The Tukey test was used as a post hoc test. All statistical analysis was performed using the OriginPro 2015 (OriginLabs) software. Weighted average values are presented with weighted standard deviation. Median values are presented with median absolute deviation.

References in Supplementary Information:^25,53^.

### Supplementary Information

Supplementary Information.

## Data Availability

Further information and requests for resources and reagents should be directed to and will be fulfilled by the corresponding authors. Materials and data generated in this study are available upon request from the corresponding authors.
