# Supplementary material for: Investigation of nano- and microdomains formed by ceramide 1 phosphate in lipid bilayers
Source: Sci Rep. 2023 Oct 30;13:18570. doi: 10.1038/s41598-023-45575-5 (PMC10616280; doi:10.1038/s41598-023-45575-5)
Supplement: Supplementary file 1 — Supplementary Information. [file 41598_2023_45575_MOESM1_ESM.docx]

**Supporting Information: Investigation of nano- and micro-domains formed by ceramide-1-phosphate in lipid bilayers**

Dominik Drabik^1,2^, Mitja Drab^3^, Samo Penič^3,4^, Aleš Iglič^3^ and Aleksander Czogalla^1^

^1^Laboratory of Cytobiochemistry, Faculty of Biotechnology, University of Wroclaw, F. Joliot-Curie 14a, 50-383 Wrocław, Poland

^2^Department of Biomedical Engineering, Faculty of Fundamental Problems of Technology, Pl. Grunwaldzki 13, 50-377, Wrocław, Poland

^3^Laboratory of Physics, Faculty of Electrical Engineering, University of Ljubljana, Tržaška cesta 25, 1000 Ljubljana, Slovenia

^4^Laboratory of Bioelectromagnetics, Faculty of Electrical Engineering, University of Ljubljana, Tržaška cesta 25, 1000 Ljubljana, Slovenia

**1. spot-variation zetaFCS on lipid vesicles**

**1.1 Z-fits and autocorrelation curves**

In this section examples of Z-fits for sv-zFCS data were presented along with corresponding autocorrelation curves. The results are presented for POPC (Fig S1), POPC:C1P18 8:2 (Fig S2) and POPC:C1P16 8:2 (Fig S3).

Fig S1. (A) Diffusion times corresponding to various z-positions (commonly referred to as z-scans) for a single POPC GUV are recorded with the waist set to 236 nm. These times are then fitted with a quadratic function to calculate the minimum diffusion time for this vesicle. (B) The corresponding autocorrelation function is employed to determine one of the diffusion times derived from the previous panel. (C) Similarly, diffusion times corresponding to various z-positions for another single POPC GUV are recorded, this time with the waist set to 282 nm. Again, these times are fitted with a quadratic function to calculate the minimum diffusion time for this vesicle. (D) As in panel B, the corresponding autocorrelation function is used to determine one of the diffusion times derived from the previous panel.

Figure S2. (A) Diffusion times for various z-positions (also known as z-scans) for a single GUV of POPC:C1P18 at a ratio of 8:2 were recorded, with the waist set to 212 nm. These were fitted with a quadratic function to calculate the minimum diffusion time for this vesicle. (B) The corresponding autocorrelation function was used to determine one of the diffusion times from the previous panel. (C) Diffusion times for various z-positions were again recorded for a single GUV of POPC:C1P18 at a ratio of 8:2, but this time with the waist set to 273 nm. These were also fitted with a quadratic function to calculate the minimum diffusion time for this vesicle. (D) The corresponding autocorrelation function was again used to determine one of the diffusion times from the previous panels.

Figure S3. (A) Diffusion times for varying z-positions are depicted for a single POPC:C1P16 8:2 GUV. These values were recorded with the waist set to 216 nm and fitted with a quadratic function, allowing for the calculation of the minimum diffusion time for this vesicle from the first (longer) population. (B) The corresponding autocorrelation function was used to determine one of the diffusion times from the previous panels. (C) For a single POPC:C1P16 8:2 GUV, diffusion times at various z-positions were recorded with the waist set to 305 nm. These were also fitted with a quadratic function, facilitating the calculation of the minimum diffusion time for this vesicle from the second (quicker) population. (D) The corresponding autocorrelation function was then used to determine one of the diffusion times from the previous panels.

**1.2 Probe-specific parameters determined from sv-zFCS study**

Table S1. Values of D_eff_ [µm^2^/s] determined for various probes incorporated into investigated lipid membranes.

| Membrane composition | NBD-Cholesterol | Atto488-DOPE | TopFluor-C1P | Weighted average |
| --- | --- | --- | --- | --- |
| POPC | 6.46±0.11 | - | - | - |
| POPC+C1P18 8:2 | 3.81±0.15 | 3.63±0.15 | 3.10±0.10 | 3.45±0.18 |
| POPC+C1P16 8:2 #1 | 4.19±0.17 | 4.00±0.15 | 4.11±0.10 | 4.10±0.04 |
| POPC+C1P16 8:2 #2 | 0.55±0.05 | - | 0.67±0.06 | 0.61±0.05 |

Table S2. Values of t_0_ [ms] determined for various probes incorporated into investigated lipid membranes.

| Membrane composition | NBD-Cholesterol | Atto488-DOPE | TopFluor-C1P | Weighted average |
| --- | --- | --- | --- | --- |
| POPC | 0.14±0.12 | - | - | - |
| POPC+C1P18 8:2 | 2.44±0.24 | 0.65±0.28 | 0.79±0.10 | 1.14±0.42 |
| POPC+C1P16 8:2 #1 | -(1.76±0.36) | -(0.53±0.16) | -(0.91±0.10) | -(0.92±0.23) |
| POPC+C1P16 8:2 #2 | 99.2±2.1 | - | 91.3±1.4 | 94.4±2.8 |

**2. 3D reconstructions of giant unilamellar vesicles**

In this section the 3D reconstructions for vesicles with POPC:C1P18 (Fig S4 A-L) and POPC:C1P16 (Fig S6 A-L) are presented. Vesicles were labelled with Atto488-DOPE, DiO and NBD-Chol. At least 3 vesicles are shown for each of the dye. Additionally, populations of circularity and roundness calculated for observed domains are presented in FigS5.

<


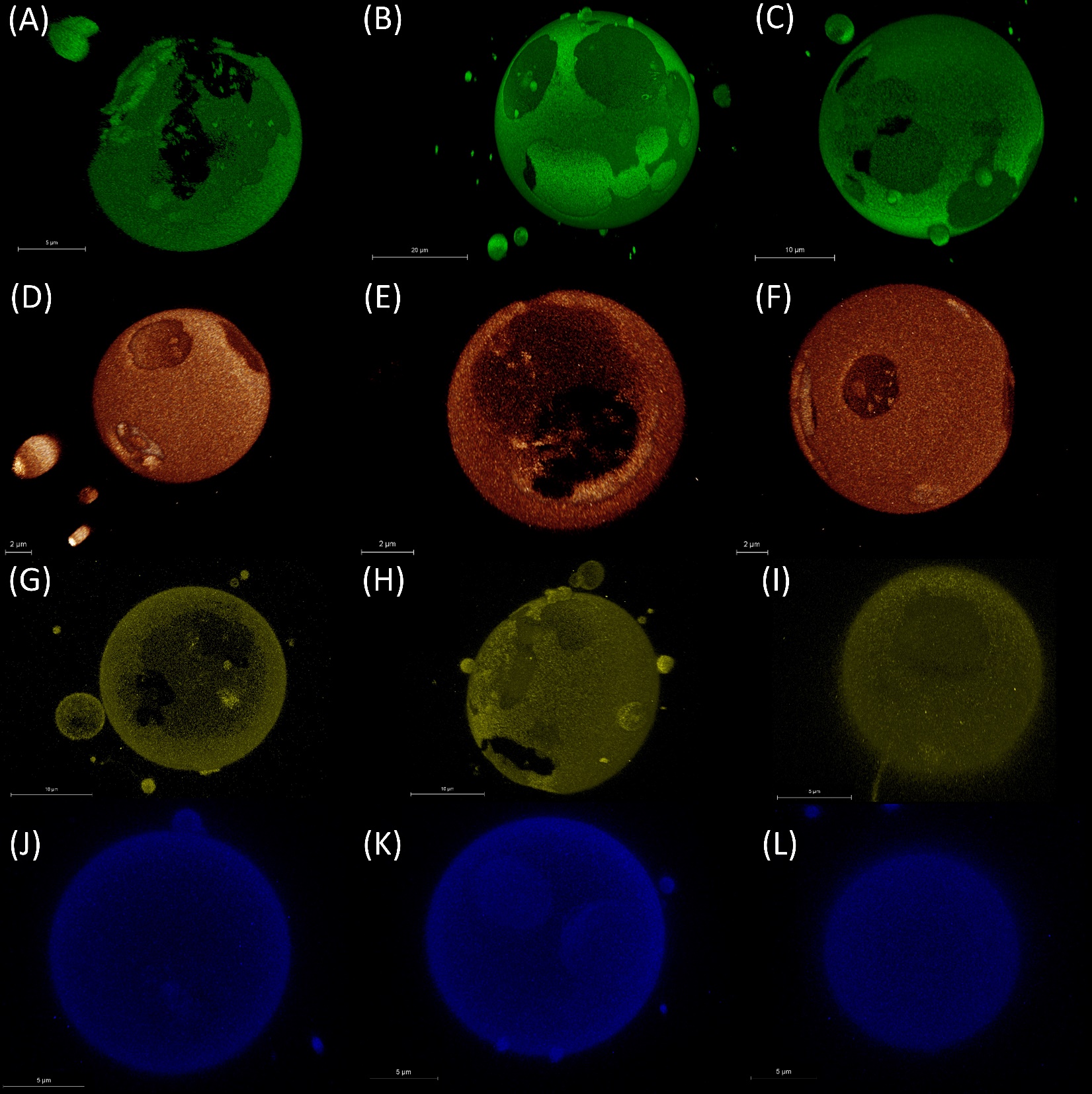


Figure S4. 3D reconstructions of POPC:C1P18 8:2 labelled with (A-C) Atto488, (D-F) DiO, (G-I) NBD-Chol and (J-L) TopFluor-C1P.

Figure S5. (A) Circularity and (B) roundness populations of experimentally determined domains for both POPC:C1P16 8:2 and POPC:C1P18 8:2 membrane compositions.


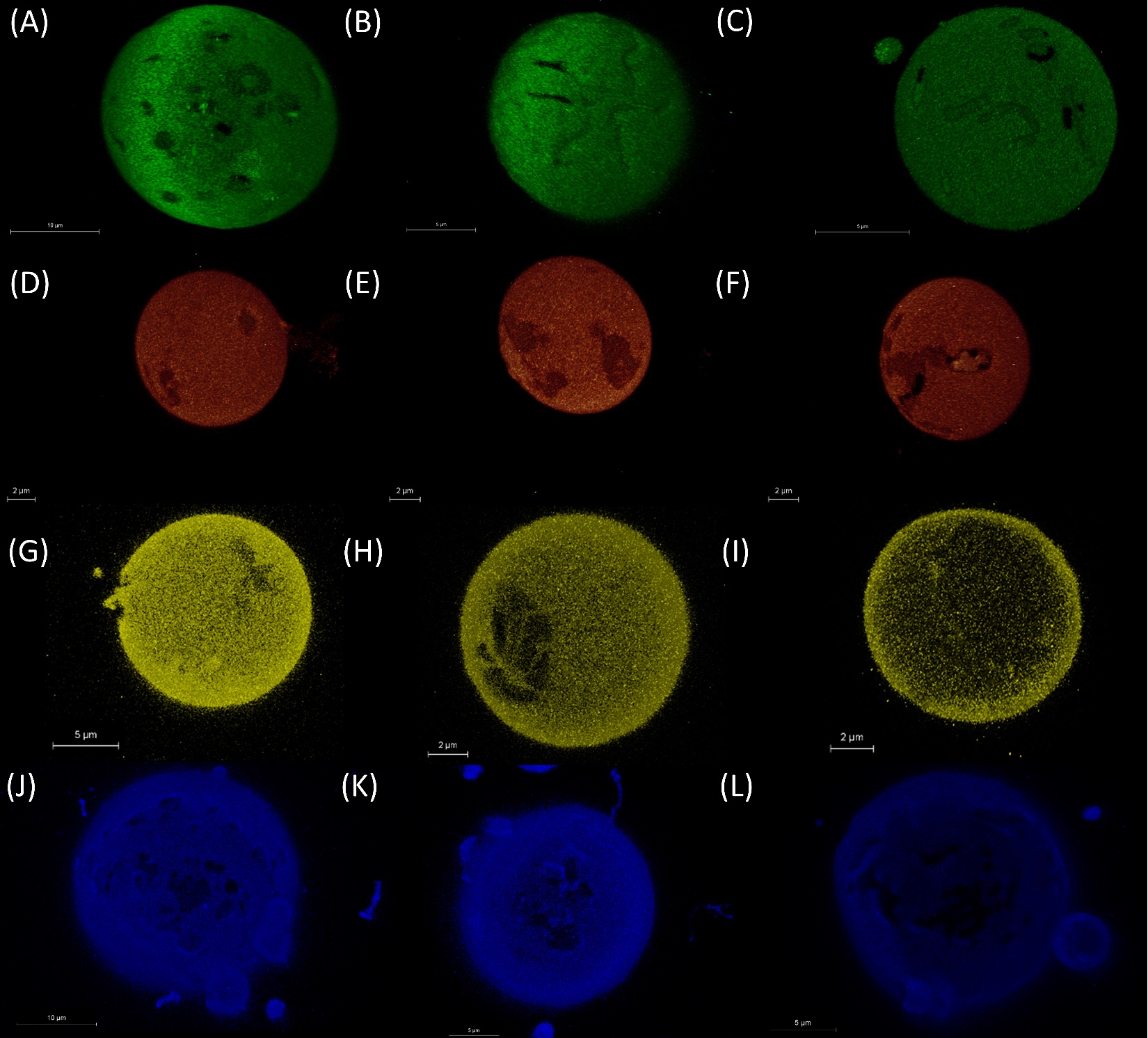


Figure S6. 3D reconstructions of POPC:C1P16 8:2 labelled with (A-C) Atto488, (D-F) DiO and (G-I) NBD-Chol and (J-L) TopFluor-C1P.

**3. FCS model fitting consideration**

Our investigation led to an intriguing outcome regarding the POPC:C1P18 membrane labeled with TopFluor-C1P, which exhibited a conspicuous absence of a second population. Distinct domains within giant unilamellar vesicles (GUVs) were not discernible in this instance, yet drawing from analogous results from other dyes, a plausible hypothesis could be that TopFluor-C1P was distributed evenly across both phases. Consequently, a dual population should be detectable in this model using svFCS studies.

However, this seeming discrepancy can be interpreted through the lens of model fitting. It's noteworthy that the discrepancy in the diffusion coefficients obtained from in silico simulations for the POPC and POPC:C1P18 systems is marginal, approximately 1 µm2/s. Figure S7.A displays two simulated autocorrelation curves, computed using OriginLab, with uniform parameters (⟨N⟩=1, V_eff_=0.2fl, structural parameter κ=3) barring the diffusion time. In the first, the diffusion time was adjusted to 3.585 ms, aligning with a diffusion coefficient of 3.65 µm^2^/s, while in the latter, it was calibrated to 2.726 ms, corresponding to a D value of 4.8 µm^2^/s. A third curve, representing a two-population model with equal population fraction, was also plotted.

These initial differences between the two models are minor. It's also worth noting that a two-population model can be easily misinterpreted as a single‑population model due to this similarity. This complexity is exacerbated when we take into account that experimental data tends to be noisy. Figure S7.B illustrates that if the level of noise is sufficiently high, it becomes challenging to differentiate between two populations. In such a scenario, where the diffusion coefficient values are closely adjacent, it could consequently lead to the fitting of a one-population model.

Figure S7. Simulated autocorrelation curves with diffusion coefficients corresponding to *in silico* results (A) without and (B) with noise.

**4. Model and simulation details**

In the present model, the energy of a lipid vesicle is expressed as the sum of contributions of membrane bending and direct interactions be-tween nano‑domains (nDs) embedded into the lipid membrane:

$$W=W_{B}+W_{I}$$

where WB is membrane bending energy and WI is interaction between C1P domains. For the membrane bending energy the standard Helfrich expression is used (1) :

$$W_{B}=\frac{ϰ}{2}\int\left( 2h-c_{0} \right)^{2} dA,$$

where the integral runs over the whole area of the membrane with bending stiffness *𝛞*, *h* is the mean curvature and *c_0_* the spontaneous curvature of membrane and C1P domains (set to zero in this work). For direct interactions between neighboring C1P domains we assume the step potential

$$W_{I}=-w\sum_{i<j} H\left( r_{0}-r_{ij} \right),$$

where *w* is a direct interaction constant, the sum runs over all C1P domain pairs, *r_ij_* are their mutual in-plane distances, *H* is the Heaviside step function and *r_0_* is the range of the direct interaction. We consider here attractive interactions w to account for the fact that C1P domains attract. The membrane is represented by a set of *N* vertices that are linked by tethers of variable length *l* to form a closed, dynamically triangulated, self-avoiding two‑dimensional network of approximately 2*N* triangles and with the topology of a sphere. The lengths of the tethers can vary between a minimal and a maximal value, l_min_, and l_max_, respectively. Self-avoidance of the network is ensured by choosing the appropriate values for l_max_ and the maximal displacement of the vertex *s* in a single updating step. One Monte-Carlo sweep (MCs) consists of individual attempts to displace each of the *N* vertices by a random increment in the sphere with radius *s*, centered at the vertex, followed by RBN attempts to flip a randomly chosen bond. We denote RB as the bond-flip ratio, which defines how many attempts to flip a bond are made per one attempt to move a vertex in one MCs. Note that the bond-flip ratio is connected to the lateral diffusion coefficient within the membrane, i.e. to the membrane viscosity. In this work we have chosen RB=3,  s/_lmin_=0.15. and l_max_/l_min_=1.7. The dynamically triangulated network acquires its lateral fluidity from a bond flip mechanism. A single bond-flip involves the four vertices of two neighboring triangles. The tether connecting the two vertices in diagonal direction is cut and reestablished between the other two, previously unconnected, vertices. The self-avoidance of the network is implemented by ensuring that no vertex can penetrate through the triangular network and that no bond can cut through another bond (2).

**5. Monte Carlo and experiment assessment for interaction energy determination**

To establish which interaction energy *w* was most accurately reflecting dynamics of domains, the data from Monte Carlo simulations was normalized to experimental data. This was followed by establishing the difference between the *in silico* and *in vitro* bars in each of the coverage areas. This is presented for POPC:C1P18 case in lower plots in Figure S8. Finally, the absolute values were summed, which resulted in accumulative residue equaled to 36.8, 35.2, 34.6, 55.2 and 64.0 for w=1.2, 1.3, 1.4, 1.6 and 1.8. Clearly, the accumulative residue decreased with increase of *w*.

**
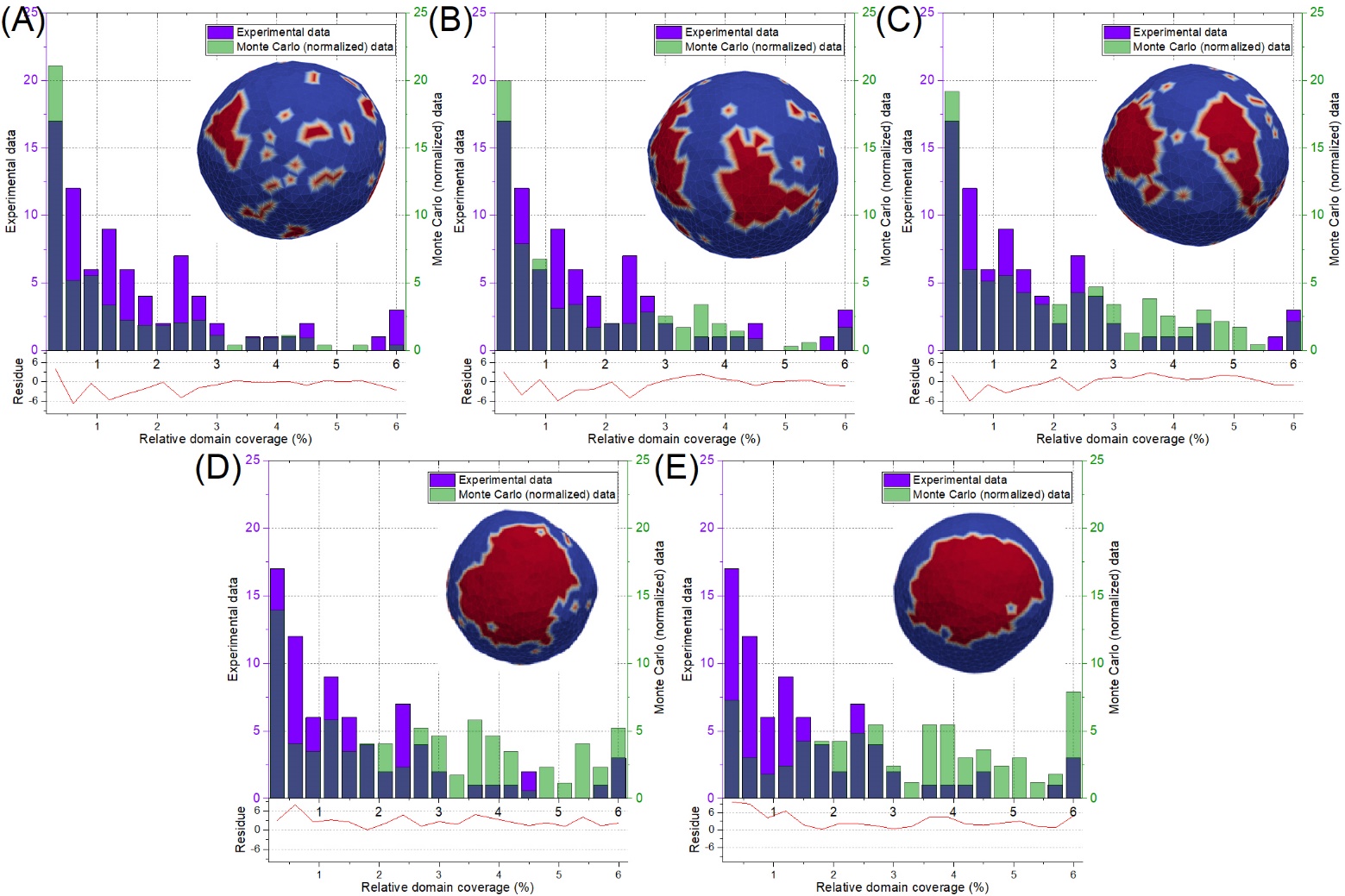
**

Figure S8. Comparison of relative domain coverage from experimental and simulation studies for POPC:C1P18 system with (A) w=1.2, (B) w=1.3, (C) w=1.4, (D) w=1.6, (E) w=1.8. Violet and green bars represent excess of either Monte Carlo or experimental data. Dark blue bars represent coverage of both sources (bars).

Similar analysis was done for POPC:C1P16:0 case, as presented in Figure S9. The absolute values were summed, which resulted in accumulative residue equaled to 12.1, 16.5, 19.2, 36.1 and 40.8 for w=1.2, 1.3, 1.4, 1.6 and 1.8. Contrary to previous C1P18, in the case of C1P16 the accumulative residue increased with increase of *w*.

**
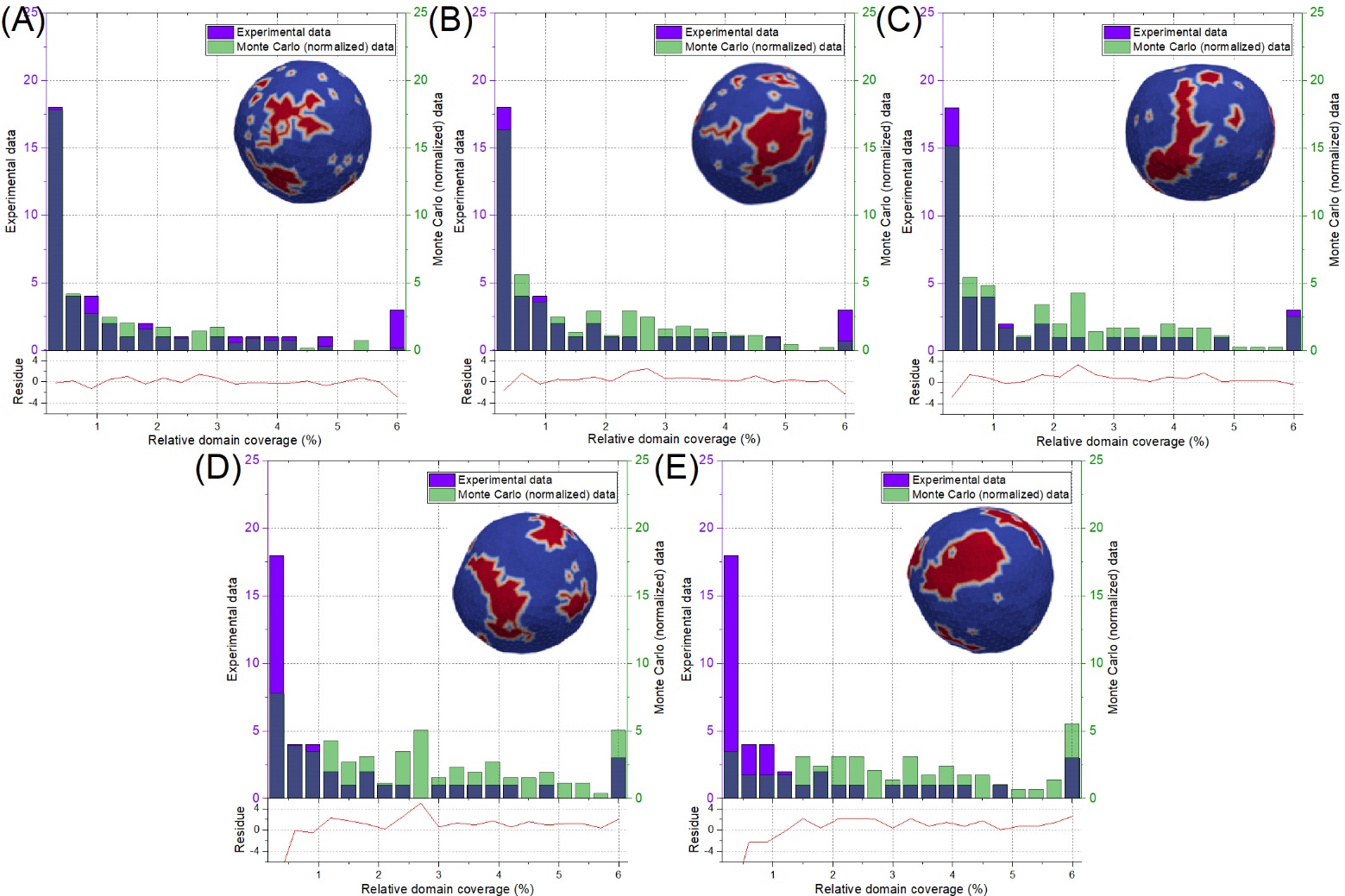
**

Figure S9. Comparison of relative domain coverage from experimental and simulation studies for POPC:C1P16 system with (A) w=1.2, (B) w=1.3, (C) w=1.4, (D) w=1.6, (E) w=1.8. Violet and green bars represent excess of either Monte Carlo or experimental data. Dark blue bars represent coverage of both sources (bars).

**
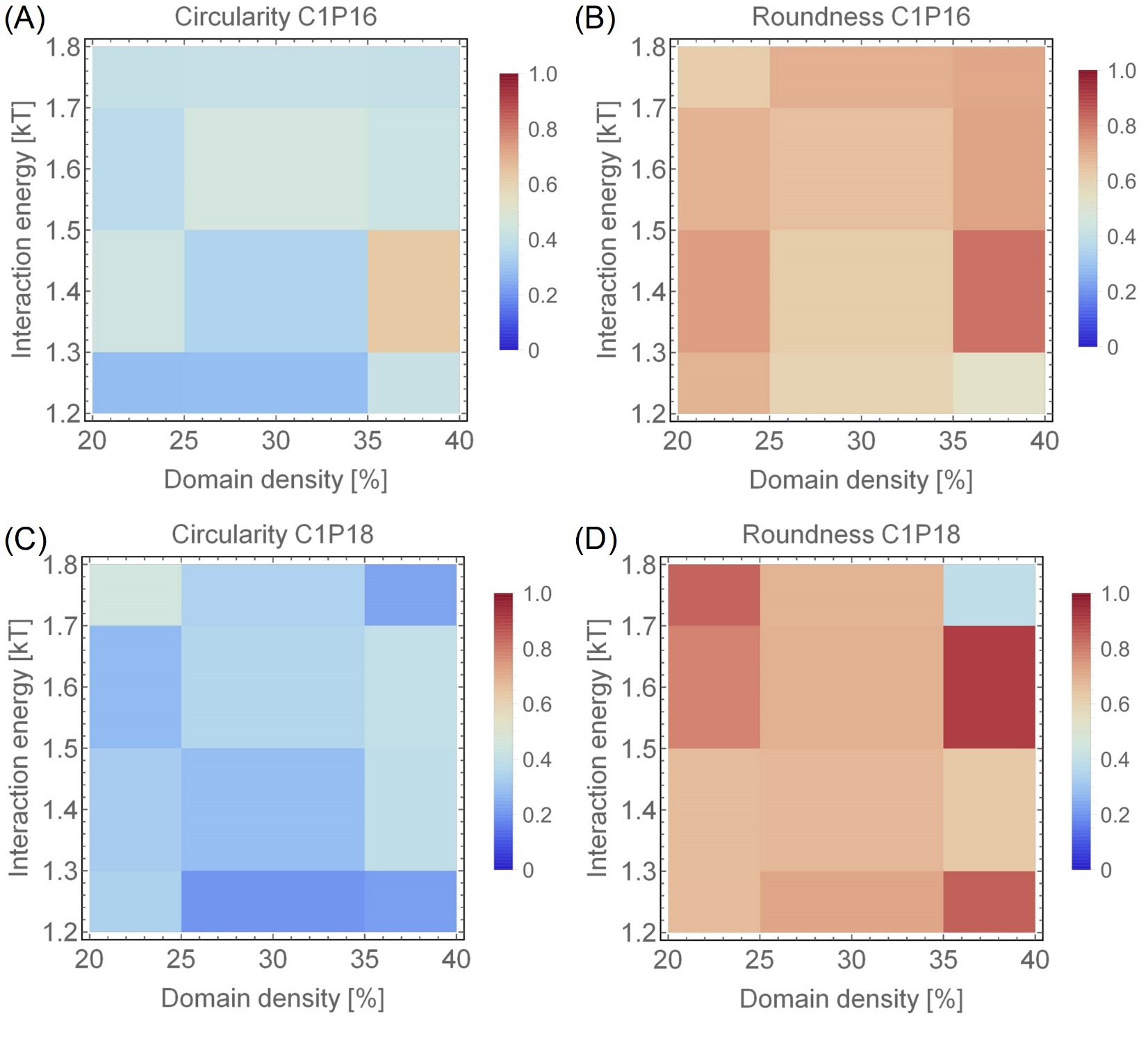
**

Figure S10. (A,C) Circularity and (B,D) roundness populations of computationally determined domains for both POPC:C1P16 (A-B) and POPC:C1P18 (C-D) membrane compositions from Monte Carlo simulations.

6. Structures of lipids


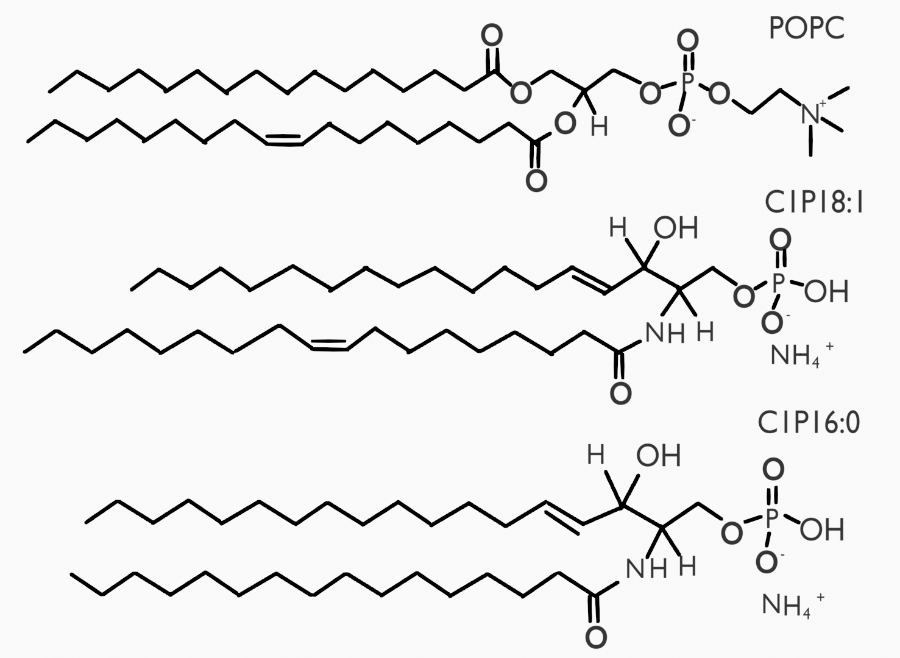


Figure S11. Structures of lipids used in the study.


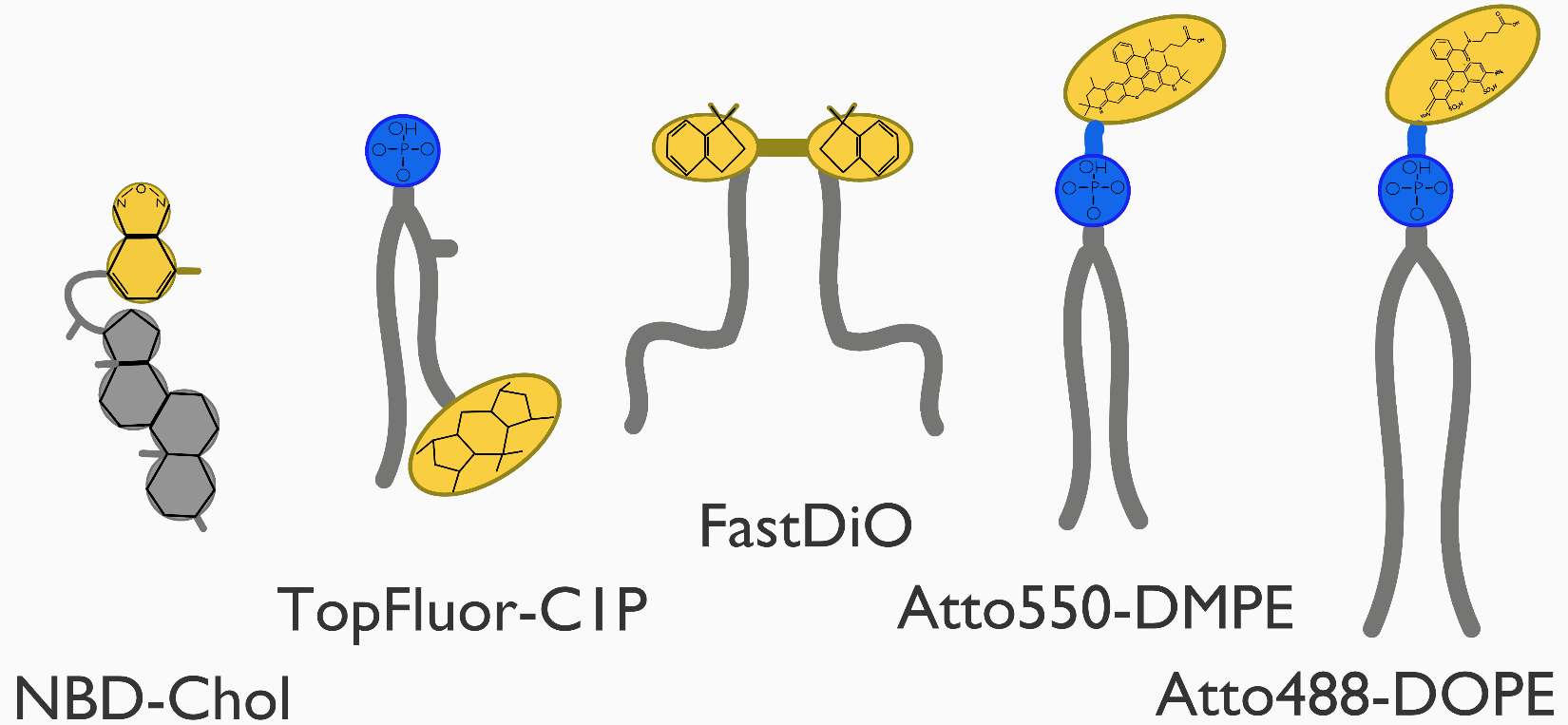


Figure S12.  Sketches of fluorophores (yellow) used in this study showing their positions within lipid analogs. Lipid phosphorus group is colored in blue.

7 Computational Details

7.1 Force field modification


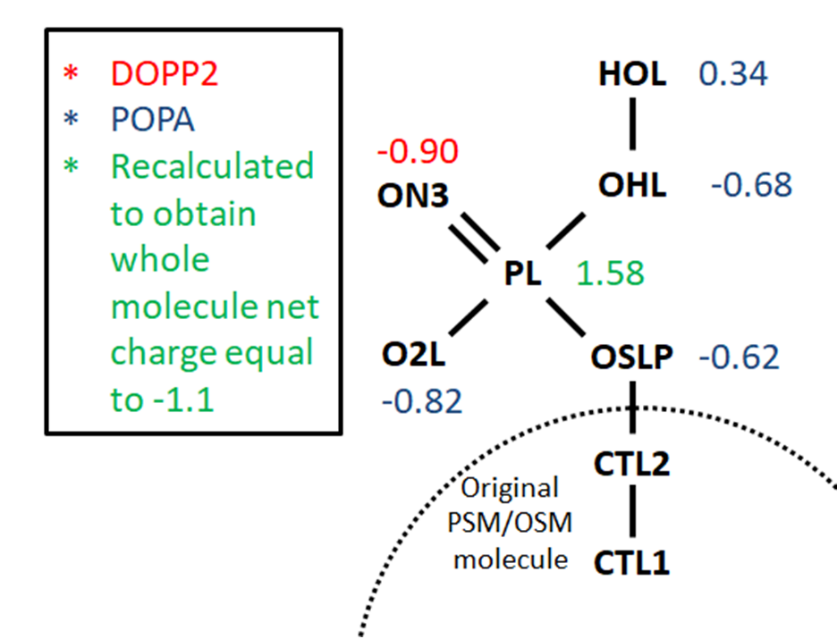


Figure S13. Detailed charge distribution of the head region of modified C1P molecule force field. Red and blue numbers represent the charges of corresponding force fields of DOPP2 and POPA while green number represent the recalculated charge to maintain original charge of lipid head.

7.2 Molecular dynamics systems


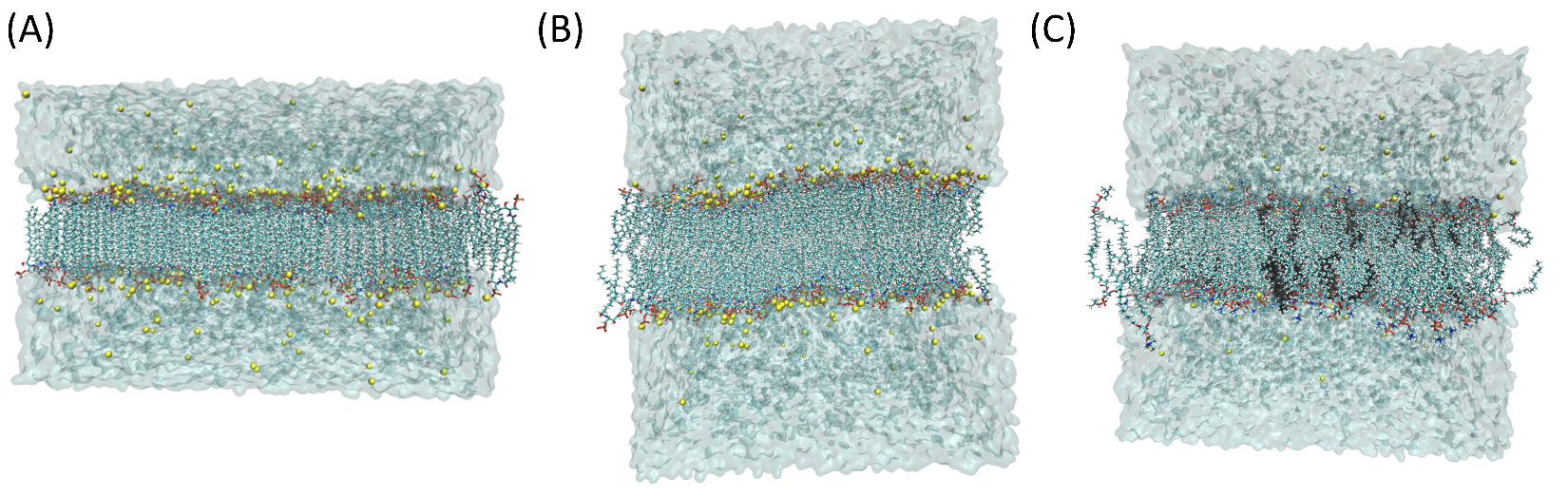


Figure S14. Snapshots of molecular dynamics systems. (A) C1P16, (B) C1P18 pure membranes. (C) POPC:C1P16 8:2 membrane (C1P colored black).

**References**

1. W. Helfrich, Elastic properties of lipid bilayers: theory and possible experiments. *Z Naturforsch C* **28**, 693-703 (1973).

2. M. Fosnaric *et al.*, Theoretical study of vesicle shapes driven by coupling curved proteins and active cytoskeletal forces. *Soft matter* **15**, 5319-5330 (2019).
